# Giant hepatoid adenocarcinoma of the transverse colon in an 84-year-old woman: A case report and review of the literature

**DOI:** 10.1097/MD.0000000000043937

**Published:** 2025-08-22

**Authors:** Chen Lin, Ziyan Wang, Peipei Wang, Qianyu Wang, Mingxia Li, Bin Wu

**Affiliations:** a Department of General Surgery, Peking Union Medical College Hospital, Chinese Academy of Medical Sciences and Peking Union Medical College, Beijing, China; b School of Medicine, Tsinghua University, Beijing, China; c Department of Orthopedics, Peking Union Medical College Hospital, Chinese Academy of Medical Sciences and Peking Union Medical College, Beijing, China.

**Keywords:** case report, colorectal cancer, hepatoid adenocarcinoma, transverse colon

## Abstract

**Rationale:**

Hepatoid adenocarcinoma (HAC) is a rare extrahepatic tumor. The most common origin of HAC was the stomach (63%). HACs originating from the colon or rectum are rare.

**Patient concerns:**

An 84-year-old Chinese woman was presenting with a large abdominal mass. Imaging revealed an irregularly thickened transverse colon wall with its greatest dimension over 15 cm. Colonoscopy identified a space-occupying lesion in the transverse colon.

**Diagnoses:**

Histopathology confirmed poorly differentiated adenocarcinoma with partial hepatoid differentiation.

**Interventions:**

The patient underwent an extended right hemicolectomy and partial small intestinal resection. Immunohistochemistry revealed mismatch repair deficiency characterized by MLH-1 and PMS-2 loss.

**Outcomes:**

The patient recovered and was discharged. At 30 months of follow-up, no recurrence or metastasis was observed.

**Lessons:**

HACs are less likely to occur in the colorectum. We recommend that surgeons intervene aggressively if the patient’s condition permits.

## 1. Introduction

Hepatoid adenocarcinoma (HAC) is a rare type of extrahepatic cancer, resembling hepatocellular carcinoma (HCC), and often producing alpha-fetoprotein (AFP). HAC was first reported as a gastric AFP-producing cancer.^[1]^ Such histological and physiological similarities make it difficult to distinguish the 2. The most common origin for HAC is the stomach region (63%).^[2–4]^ Clinically, HAC is considered more aggressive and has a worse prognosis than common adenocarcinomas, partly due to its high metastatic potential and the lack of standardized treatment strategies. In recent years, minimally invasive surgical approaches have shown promise in improving perioperative outcomes in selected patients, though their role in HAC remains to be fully defined. HACs originating from the colon or rectum are rare. To the best of our knowledge, there have been no known reported cases of HAC measuring over 15 cm in greatest dimension. Hence, we report a case of an 84-year-old woman with a large abdominal mass admitted to Peking Union Medical College Hospital. The patient was diagnosed with HAC of the transverse colon, which was confirmed by postoperative pathological examination. This is the first report of a surgically treated enlarged HAC of the colon. In addition, we reviewed the literature on HAC of the colon or rectum.

## 2. Case description

**Figure 1.**
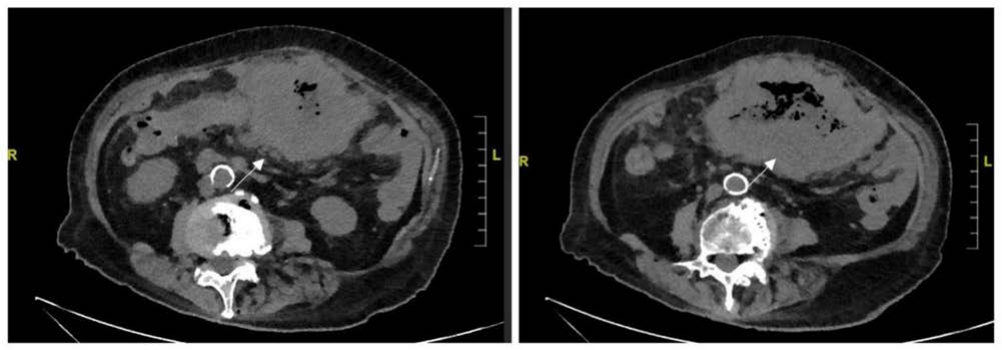
The CT plain scan revealed an irregularly thickened transverse colon wall. The mass was larger than 15 cm (arrows).

**Figure 2.**
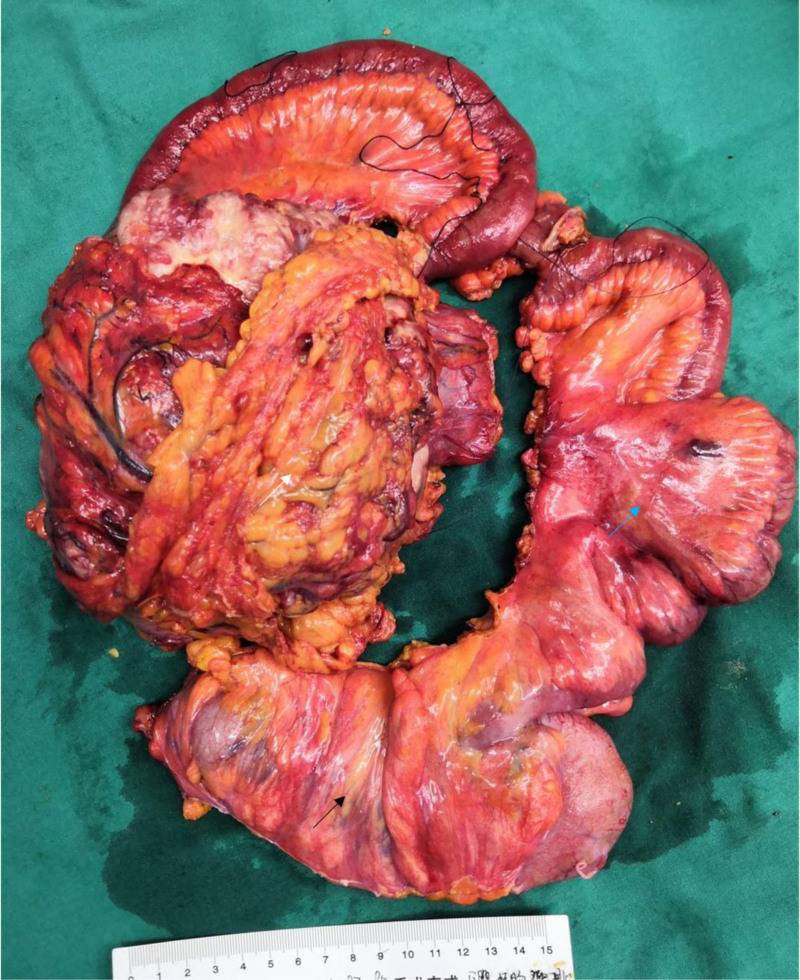
Surgical specimens of the patient who underwent an extended right hemicolectomy (blue arrow) with tumor (white arrow) and partial small intestinal (black arrow) resection are seen.

**Figure 3.**
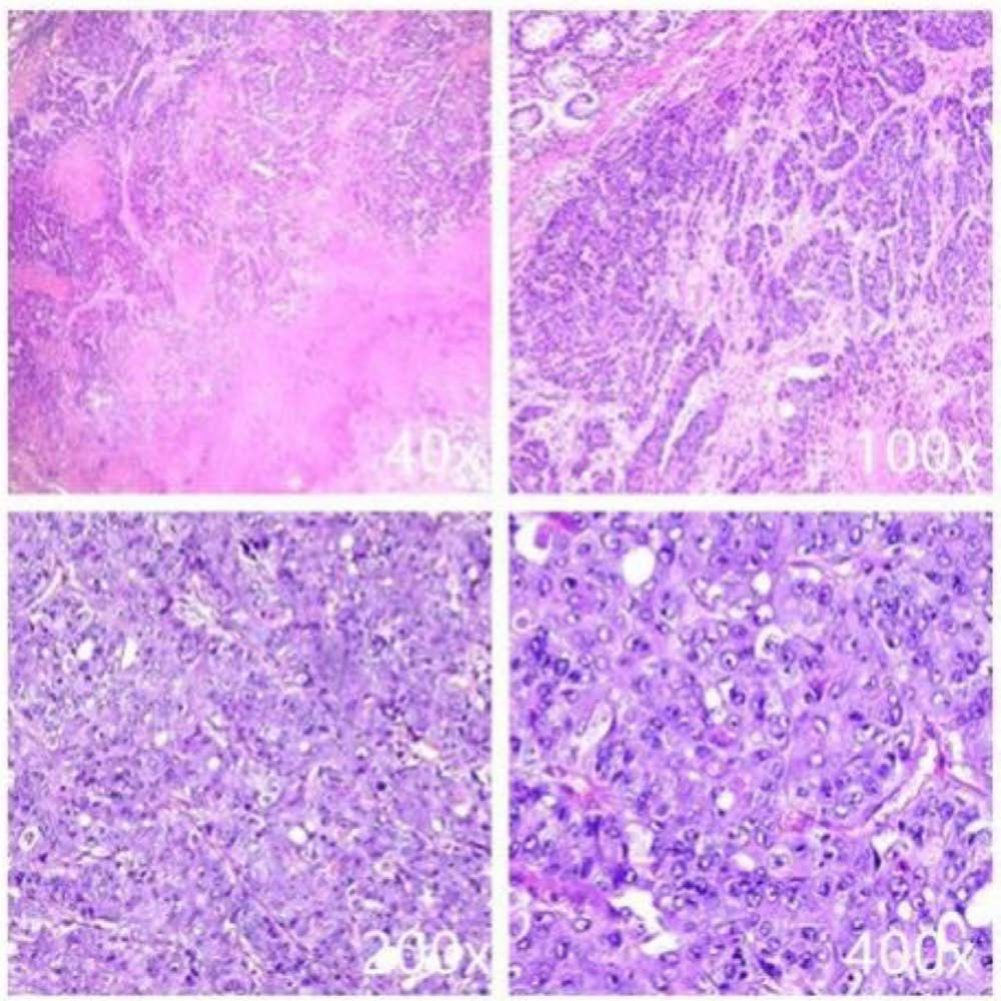
The hematoxylin–eosin staining shows poorly differentiated adenocarcinoma of the colon, partially hepatoid adenocarcinoma.

**Figure 4.**
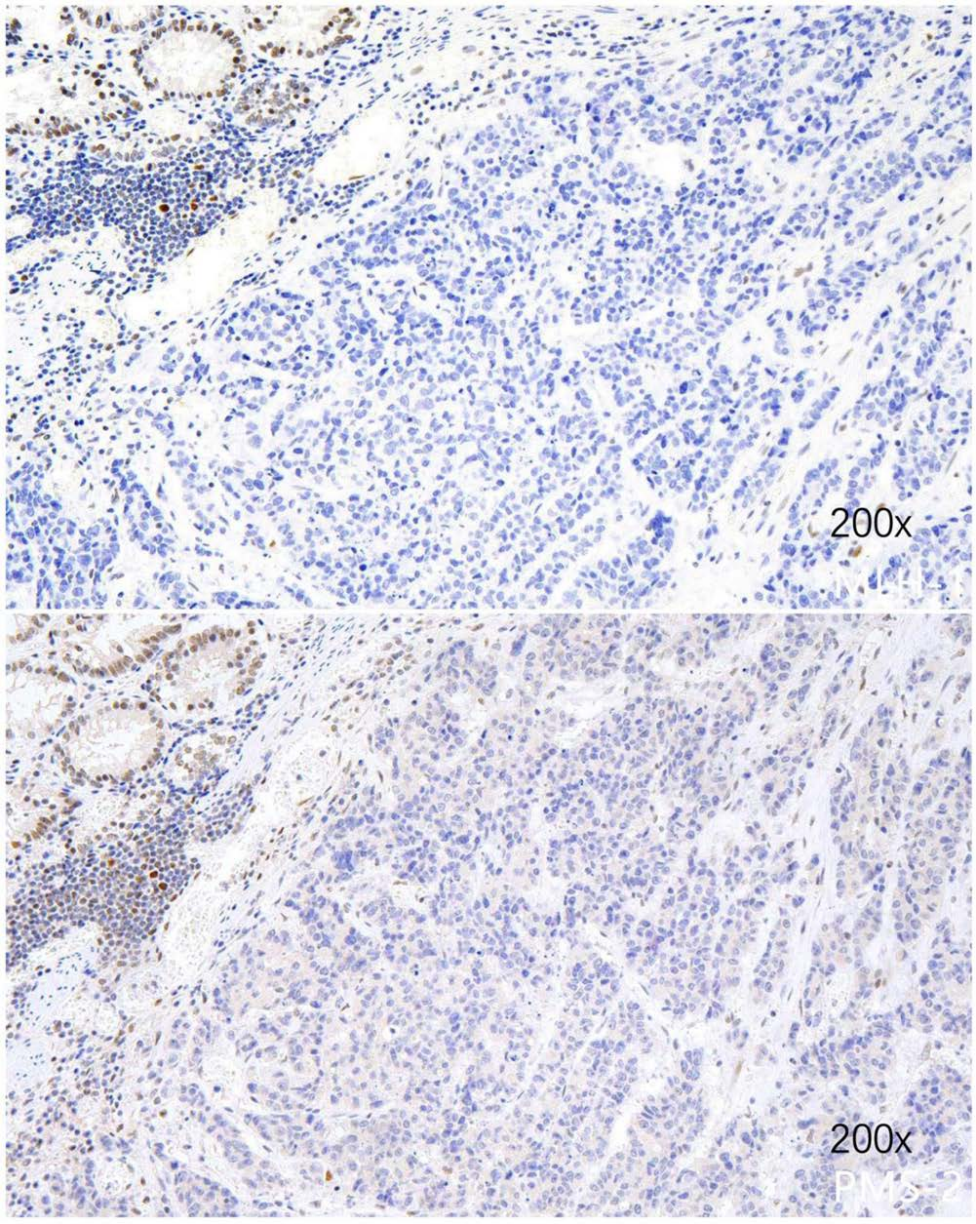
Immunohistochemical analysis reveals negative MLH-1 and PMS-2.

An 84-year-old Chinese woman presented to the clinic with a palpable, clearly defined, and mobile mass on physical examination. A plain CT scan due to contrast allergy revealed irregularly thickened transverse colonic walls, approximately 4.5 cm thick and over 15 cm in its greatest dimension. The serosal surface appeared rough, and multiple nodular shadows were noted in the surrounding tissues (Fig. 1). A colonoscopy performed 1 month prior to admission revealed an ulceroinfiltrative lesion in the transverse colon, along with diverticula and polyps in the sigmoid, excluding ulcerative colitis. Biopsy confirmed adenocarcinoma, with elevated serum carcinoembryonic antigen level (48.4 ng/mL, normal < 5 ng/mL), while CA199 was normal (3.5 U/L), AFP was not measured. The patient had no history of smoking, alcohol consumption, hepatitis, liver fibrosis, inflammatory bowel disease, or cirrhosis. The patient had no family history of similar diseases or tumors. There was no evidence of distant metastases to the liver or lungs. Furthermore, extended right hemicolectomy and partial small intestinal resection were performed, and postoperative pathology confirmed a mixed form of neoplasia: moderately to poorly differentiated adenocarcinoma of the colon with focal hepatoid differentiation (Fig. 2). Pathological analysis indicated infiltration through the intestinal wall and surrounding adipose tissue (Fig. 3). Moreover, histological analysis of the adjacent lymph nodes revealed chronic inflammation (0/22). Immunohistochemical analysis revealed positive results for MSH-2 and MSH-6 but negative results for MLH-1 and PMS-2 (Fig. 4). The patient recovered well postoperatively, without complications, and was discharged on postoperative day 7. Due to her age and the tumor’s pathological characteristics, she did not undergo postoperative chemotherapy. Normal serum levels and CT scans showed no evidence of recurrence at the 30-month follow-up.

## 3. Discussion

HAC has been reported in multiple organs including the stomach,^[1,2]^ lungs,^[5,6]^ kidneys,^[7]^ gallbladder,^[8]^ peritoneum, and omentum.^[9]^ The most common origin of HACs is the stomach, and they share similar histological and clinical features with those of the colon or rectum.^[10]^ A recent study summarized 39 cases of colorectal HACs reported over the last 30 years.^[11]^ In the colon or rectum, 46.2% were located in the right hemicolon, 25.6% in the left hemicolon, and 23.1% in the rectum.^[11,12]^ There has been a male predominance (male:female = 2.4:1) in patients with HAC,^[4]^ which is consistent with the gender distribution observed in colorectal tumors (2:1).^[3,11]^ Moreover, HACs in the colon and rectum have unique features such as blood in the stool and abdominal pain.^[11]^ Additionally, a few cases of HAC have been reported in patients with ulcerative colitis.^[13–15]^ However, whether ulcerative colitis induces HAC requires further investigation.

HACs present with symptoms, such as abdominal distension, pain, and melena, similar to intestinal cancers. Elevated serum alpha-fetoprotein (AFP) levels, a hallmark of HACs, help in their detection, although they are not always present, particularly in colon and rectal cases. While helpful, AFP levels are not specific to HACs because adenocarcinomas in these regions can also produce AFP.^[16]^ The aggressive nature of HACs underscores their propensity for recurrence and metastasis, especially to the lymph nodes and liver, with AFP levels correlating with metastasis risk and vascular endothelial growth factor influencing tumor angiogenesis.^[17]^ In our case, AFP was not measured at the time of diagnosis, as our preoperative workup primarily considered the lesion to be a conventional colon carcinoma, and this omission highlights the need for a more comprehensive differential diagnosis in cases of unusually large colonic tumors.

Molecularly, HACs share markers with HCC, such as glypican-3,^[9,18,19]^ AFP, and SALL4,^[18]^ which aid in differential diagnosis. Originating from the endodermal layers, both colonic and hepatic tissues can produce AFP, indicating their embryonic commonality and explaining AFP’s role in the diagnosis of HAC. However, the diagnostic utility of AFP is limited because some patients with HAC may not show elevated AFP levels associated with tumor differentiation.^[20,21]^ These adenocarcinomas, possessing morphological features resembling HCC but do not produce AFP, are called AFP-negative HACs. It has been reported that AFP-negative HAC often produces other hepatoid substances, such as albumin, α-antitrypsin, and α-chymotrypsin.^[19]^ Hep-1 is another hepatocyte marker that is not expressed in other tumors. Hep-1 positive cells are the most specific and sensitive markers of hepatocyte differentiation. The sensitivity and specificity of hep-1 for diagnosing HCC are 80% and 90%,^[22,23]^ respectively. The combination of AFP and Hep-1 improves diagnostic accuracy for HAC.

Current treatments align with those for intestinal adenocarcinomas with an emphasis on surgical resection, chemoradiotherapy, and palliative care. Radical surgery has been shown to improve survival with chemotherapy regimens, including FOLFOX,^[3,15,24,25]^ FOLFIRI with bevacizumab, and cetuximab for specific genetic profiles. The prognosis for patients with HAC remains poor, particularly in advanced stages, highlighting the need for tailored therapeutic strategies based on HAC’s unique molecular and clinical characteristics. Given the advanced age of our patient and the significant tumor burden, nutritional support played an important role in her perioperative management. Preoperative nutritional evaluation and intervention have been shown to reduce surgical complications and improve recovery, especially in elderly oncologic patients.^[26]^

In our case, the patient had a dMMR tumor indicated by negative MLH-1 and PMS-2 on immunohistochemical analysis. Literature elucidates the relationship between HAC and dMMR/MSI,^[27]^ particularly in gastrointestinal and pancreatobiliary districts. Molecular profiling of gastrointestinal hepatoid tumors revealed common alterations like TP53 mutations, MSI, and Her-2 amplification, suggesting MSI’s significant role in HAC’s pathogenesis and progression. Investigations have suggested that MSI may be a candidate for immune checkpoint blockade therapies owing to the observed enrichment of tumor-infiltrating lymphocytes in tumors. However, 1 limitation of this case is that immunohistochemical staining for hepatoid-specific markers, such as glypican-3, SALL4, and HepPar1, was not performed, which may limit the pathological confirmation of hepatoid differentiation. The immunohistochemical evaluation was limited to MMR proteins and HER-2, and FISH testing for EBV (EBER) was negative. Additionally, the patient did not undergo MSI/MSS molecular testing or receive any postoperative antitumor therapy. Given her history and absence of prior colorectal disease, her dMMR tumor likely resulted from a somatic mutation. In conclusion, HACs are less likely to occur in the colorectal region, and such enlarged tumors are rare. A literature review suggests that the presence of these tumors may indicate a poor prognosis. First, 84.6% of patients were classified as stage III–IV, and nearly half had distant metastases, primarily in the liver.^[3]^ Second, the reported median survival for patients was 8 months, with a 1-year overall survival rate of 31.0%,^[11]^ and significant prognostic factors include depth of invasion, liver metastasis, TNM stage, and complete resection.^[11]^ Therefore, surgeons should aggressively intervene if the patient’s condition permits intervention. Although our patient was 84 years old, she recovered well postoperatively. This case report can be used as a reference by clinicians when treating patients with HACs.

## Author contributions

**Conceptualization:** Chen Lin, Ziyan Wang, Peipei Wang, Qianyu Wang, Mingxia Li, Bin Wu.

**Writing – original draft:** Ziyan Wang, Peipei Wang.

**Writing – review & editing:** Ziyan Wang, Peipei Wang.

**Investigation (literature review):** Chen Lin, Mingxia Li.

**Investigation (surgery):** Bin Wu.
